# Why CCR2 and CCR5 Blockade Failed and Why CCR1 Blockade Might Still Be Effective in the Treatment of Rheumatoid Arthritis

**DOI:** 10.1371/journal.pone.0021772

**Published:** 2011-07-01

**Authors:** Maria C. Lebre, Clarissa E. Vergunst, Ivy Y. K. Choi, Saïda Aarrass, Ana S. F. Oliveira, Tim Wyant, Richard Horuk, Kris A. Reedquist, Paul P. Tak

**Affiliations:** 1 Division of Clinical Immunology and Rheumatology, Academic Medical Center/University of Amsterdam, Amsterdam, The Netherlands; 2 Millennium Pharmaceuticals, Cambridge, Massachusetts, United States of America; 3 Department of Pharmacology, University of California Davis, Davis, California, United States of America; Massachusetts General Hospital and Harvard Medical School, United States of America

## Abstract

**Background:**

The aim of this study was to provide more insight into the question as to why blockade of CCR1, CCR2, and CCR5 may have failed in clinical trials in rheumatoid arthritis (RA) patients, using an *in vitro* monocyte migration system model.

**Methodology/Principal Findings:**

Monocytes from healthy donors (HD; n = 8) or from RA patients (for CCR2 and CCR5 antibody n = 8; for CCR1 blockade n = 13) were isolated from peripheral blood and pre-incubated with different concentrations of either anti-CCR1, anti-CCR2, or anti-CCR5 blocking antibodies (or medium or isotype controls). In addition, a small molecule CCR1 antagonist (BX471) was tested. Chemotaxis was induced by CCL2/MCP-1 (CCR2 ligand), CCL5/RANTES (CCR1 and CCR5 ligand), or by a mix of 5 RA synovial fluids (SFs), and cellular responses compared to chemotaxis in the presence of medium alone. Anti-CCR2 antibody treatment blocked CCL2/MCP-1-induced chemotaxis of both HD and RA monocytes compared to isotype control. Similarly, anti-CCR5 antibody treatment blocked CCL5/RANTES-induced chemotaxis of RA monocytes. While neither CCR2 nor CCR5 blocking antibodies were able to inhibit SF-induced monocyte chemotaxis, even when both receptors were blocked simultaneously, both anti-CCR1 antibodies and the CCR1 antagonist were able to inhibit SF-induced monocyte chemotaxis.

**Conclusions/Significance:**

The RA synovial compartment contains several ligands for CCR1, CCR2, and CCR5 as well as other chemokines and receptors involved in monocyte recruitment to the site of inflammation. The results suggest that CCR2 and CCR5 are not critical for the migration of monocytes towards the synovial compartment in RA. In contrast, blockade of CCR1 may be effective. Conceivably, CCR1 blockade failed in clinical trials, not because CCR1 is not a good target, but because very high levels of receptor occupancy at all times may be needed to inhibit monocyte migration in vivo.

## Introduction

Rheumatoid arthritis (RA) is a chronic inflammatory disease characterized by massive infiltration of synovial tissue and synovial fluid (SF) with immune cells, mediated by chemokines and adhesion molecules [1], [2]. It is well accepted that monocyte/macrophage numbers are increased in clinically affected joints and these numbers correlate with the clinical signs and symptoms [3]. Accordingly, clinical improvement after effective antirheumatic therapy is consistently associated with reduced macrophage numbers in the synovium [4]. Taken together, synovial macrophages are considered key effector cells in the pathogenesis of RA [5], [6].

Chemokines play an important role in the accumulation of these cells at the site of inflammation. They belong to a superfamily of small (6–14 kDa) structurally related proteins that regulate the traffic of various leukocytes [7]. Inflammatory chemokines are expressed in inflamed tissues by resident and infiltrated cells upon stimulation by pro-inflammatory mediators present *in situ*. RA synovial tissue and fluid contain high concentrations of a variety of inflammatory chemokines [1], [8]–[13] that can interact with chemokine receptors on the surface of monocytes/macrophages and contribute to their accumulation at these sites. Specifically, CCR1 (major ligands CCL3/MIP-1α, CCL5/RANTES and CCL7/MCP-3), CCR2 (major ligand CCL2/MCP-1), CCR5 (ligands CCL3/MIP-1α, CCL5/RANTES and CCL7/MCP-3) are abundantly expressed by RA monocytes/macrophages [1], [10] suggesting that interference with the migration of these cells by cytokine receptor blockade might be a successful therapeutic approach to reduce synovial inflammation. Although CCR2 [14] and CCR5 [15] receptor blockade has shown positive results in animal models of RA, targeted CCR2 [16] and CCR5 [17], [18] blockade was not effective in RA patients. *In vivo* and *in vitro* experiments in RA models have also suggested that blocking CCR1 ligands or the receptor itself may inhibit chemotaxis and reduce synovial inflammation [13], [19], [20]. The experience in RA patients has been variable. The first study testing the effects of chemokine receptor blockade in human patients was a small phase 1 b proof-of-concept clinical trial in RA patients [21]. This study demonstrated evidence of a significant biological effect of a CCR1 antagonist in subjects with RA, associated with a trend towards clinical improvement. Other studies evaluating CCR1 blockade in RA have however shown no efficacy [22], [23]. To provide more insight into the question as to why these approaches might have failed, we investigated the effect of specific CCR1, CCR2 or CCR5 blockade on RA monocyte migration in an *in vitro* model evaluating SF-induced chemotaxis.

## Methods

### Ethical approval

This study was conducted with the approval of the Medical Ethical Committee of the Academic Medical Center/University of Amsterdam and all patients gave their written informed consent.

### Patients

Peripheral blood was obtained from RA patients [24] with active disease, defined by the presence of at least one clinically inflamed joint (for CCR2 or CCR5 antibodies n = 8; for CCR1 blockade n = 13 in total) and healthy subjects (n = 8). None of the patients was being treated with biologicals. Patient demographic and clinical features are shown in Table 1.

**Table 1:** Demographic and clinical data of patients (chemotaxis).

	Anti-CCR2	Anti-CCR5	CCR1 blockade
Sex, female/male (n)	8/0 (8)	7/1 (8)	5/8 (13)
Age in years, mean (range)	56.1 (44–72)	57.1 (41–78)	60.2 (40–81)
Disease duration, mean (range)	35.5 (2–108)	46 (4–120)	52.8 (1–232)
Rheumatoid factor positive, n (%)	4 (50%)	7 (87.5%)	10 (76.9%)
ACPA positive, n (%)	4 (50%)	6 (75%)	13 (90.9%)
SJC, mean (range)	6.7 (0–13)	1.6 (0–3)	5.0 (1–11)
TJC, mean (range)	8.5 (0–15)	5.8 (0–15)	7.6 (1–24)
ESR mm/h , mean (range)	21.6 (7–62)	32.7 (5–110)	18.4 (2–43)
CRP mg/liter, mean (range)	6.7 (1–21.7)	3.6 (2–4.8)	10 (0.6–34.4)

ACPA, anti-citrullinated protein/peptide antigens; SJC, swollen joint count; TJC, tender joint count; ESR, erythrocyte sedimentation rate; CRP, C reactive protein.

### Synovial fluid samples

Synovial fluid (SF) from patients with RA were collected during therapeutic arthrocentesis and transferred to heparin containing tubes. The samples were centrifuged and the supernatants stored at −80°C until used for the chemotaxis assay. Patient demographic and clinical features are shown in Table 2.

**Table 2:** Demographic and clinical data of patients (synovial fluids).

Sex, female/male (n)	3/2 (5)
Age in years, mean (range)	52.8 (32–63)
Disease duration, mean (range)	217.8 (4–692)
Rheumatoid factor positive, n (%)	4 (80%)
ACPA positive, n (%)	3 (60%)
SJC, mean (range)	4 (1–9)
TJC, mean (range)	6 (1–15)
ESR mm/h , mean (range)	39.2 (16–58)
CRP mg/liter, mean (range)	26.2 (2.8–65.5)

ACPA, anti-citrullinated protein/peptide antigens; SJC, swollen joint count; TJC, tender joint count; ESR, erythrocyte sedimentation rate; CRP, C reactive protein.

### Multiplex assay for chemokine/cytokine measurement

SFs were analyzed using the Human Cytokine Luminex 27-plex (BioSource; Invitrogen, Breda, The Netherlands). All reagents were provided with the BioSource (Invitrogen) kit, and the assay was performed according to the manufacturer's instructions.

### Monocyte isolation

Peripheral blood mononuclear cells (PBMC) were isolated by Ficoll gradient as previously described [25]. Monocytes were purified by negative selection using Monocyte Isolation Kit II (Miltenyi Biotec, Bergisch Gladbach, Germany) according to the manufacturer's instructions. The purified cells were >95% pure as determined by FACS analysis. Isolated cells were phenotyped using anti-CD3-FITC (BD Biosciences, Oxford, United Kingdom), CD14-APC (BD Biosciences), and anti-CCR2-PE (R&D systems, Abingdon, United Kingdom), anti-CCR5-PE (BD Biosciences) or anti-CCR1-PE (R&D systems) conjugated antibodies.

### Neutralizing antibodies, isotype controls and small molecule CCR1 antagonist

The following neutralizing antibodies were a gift from Millennium Pharmaceuticals Inc.: mouse anti-human CCR2 (mouse IgG2a; clone m1D9) and mouse anti-human CCR5 (mouse IgG1; clone 2D7). The neutralizing antibody against CCR1 (mouse IgG1; clone 141-2) was purchased from MBL International (Woburn, MA). Functional grade mouse IgG1 and mouse IgG2a antibodies were used as isotype controls (both from eBioscience, San Diego, CA) for CCR1/CCR5 and CCR2, respectively. The small molecule CCR1 antagonist BX471 was obtained from former Berlex Biosciences (Richmond, CA).

### *In vitro* chemotaxis

Monocytes were first washed in chemotaxis medium (PBS with 1% low endotoxin albumin, Sigma-Aldrich, Zwijndrecht, The Netherlands), incubated for 30 minutes in the absence or in the presence of various concentrations of anti-CCR antibodies (anti-CCR1: 1, 5 or 25 µg/ml; anti-CCR5: 1 or 5 µg/ml; anti-CCR2: 1, 5 or 25 µg/ml) or respective isotype controls (5 or 25 µg/ml) or with the small molecule CCR1 antagonist BX471 (1, 5 or 25 µg/ml). After incubation, 1×10^5^ monocytes were transferred into the upper chamber of 5 µM pore-size transwell plates (96 well ChemoTX®, NeuroProbe, Gaithersburg, MA). Chemotaxis medium was added to the lower chamber together with recombinant chemokines CCL2/MCP-1 (100 ng/ml; R&D systems) or CCL5/RANTES (500 ng/ml; Peprotech, Rocky Hill, NJ) or pooled RA SF (n = 5 patients, 50% diluted in chemotaxis medium). After 2 hours at 37°C, migration was quantified by staining the cells that were attached to the membrane. Briefly, after aspiration and removal of the cells from the top wells the membrane was fixed in pre-chilled methanol (bottom side down) followed by addition of DAPI solution to the membrane. After the membrane was dried, it was mounted on an OptiPlate (bottom side up) and the number of DAPI positive cells (cells that were trapped in the membrane  =  migrated cells) was quantified using a multi-label reader Victor3™ (PerkinElmer, Inc., Waltham, MA). The DAPI counts of empty wells (no addition of cells; background) were subtracted from the wells containing cells. Data are expressed as mean ± SEM of migrated cells [counts (DAPI)].

### Statistical analysis

Differences between groups were determined by unpaired t-test using the program GraphPad Prism (version 4). P values <0.05 were considered statistically significant.

## Results

### Chemokine levels in synovial fluid samples

The levels of CCR1, CCR2, and CCR5 ligands in the SFs were [mean (range) ng/ml]: CCL2/MCP-1: 4.4 (1.0–9.0), CCL3/MIP-1α: 5.6 (3.1–8.1), CCL4/MIP-1β: 9.4 (6.0–13) and CCL5/RANTES: 2.8 (0.5–4.8).

### Anti-CCR2 antibody treatment blocks CCL2/MCP-1-induced but not SF-induced HD and RA monocyte migration

**Figure 1:**
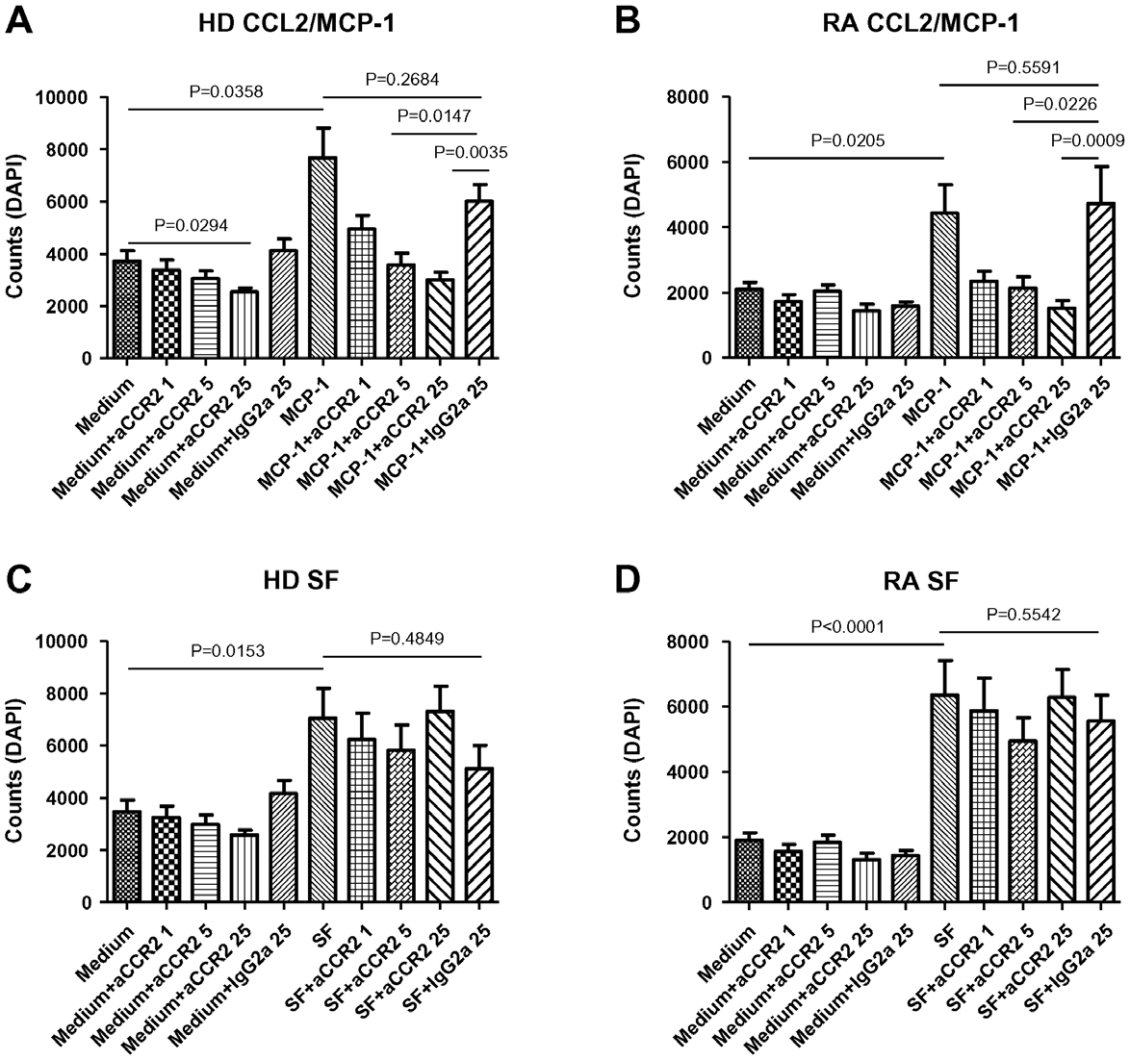
Anti-CCR2 blocks CCL2/MCP-1- but not SF-induced HD or RA monocyte migration. (A) HD monocyte migration induced by CCL2/MCP-1. (B) HD monocyte migration induced by SF. (C) RA monocyte migration induced by CCL2/MCP-1. (D) RA monocyte migration induced by SF. Data are expressed as mean ± SEM (HD n = 8; RA n = 8).

As expected, CCL2/MCP-1 induced significant migration of both HD (Fig 1A) and RA (Fig. 1B) monocytes pre-incubated with medium (HD P = 0.0358; RA P = 0.0205) or isotype control (HD P = 0.0483; RA P = 0.0005). Monocyte pre-incubation with anti-CCR2 antibodies resulted in blockade of CCL2/MCP-1-induced migration of cells derived from both HD (Fig. 1A; 5 or 25 µg/ml, P = 0.0147 and P = 0.0035, respectively) and RA patients (Fig. 1B; 5 and 25 µg/ml, P = 0.0226 and P = 0.0009, respectively). There was no effect of CCR2 blockade on spontaneous migration, except for HD monocytes at the highest antibody concentration (Fig. 1A; P = 0.0294).

Since in RA circulating monocytes are recruited to the synovial compartment under the influence of chemotactic agents, we mimicked this situation by using SF as chemoattractant in our *in vitro* model. SF induced significant migration of both HD (Fig 1C) and RA (Fig. 1D) monocytes pre-incubated with medium (HD P = 0.0153; RA P<0.0001 compared to migration in medium control groups). Contrary to results for CCL2/MCP-1-induced migration, SF-induced migration could not be blocked by anti-CCR2 antibody treatment at any of the concentrations used.

The observation that monocytes from HD showed higher chemotaxis compared to RA monocytes is not completely understood, but might perhaps be explained by the fact that RA monocytes exhibit lower expression levels for chemokine receptors compared to HD, as previously shown [1].

### Anti-CCR5 antibody treatment blocks CCL5/RANTES-induced but not SF-induced RA monocyte migration

**Figure 2:**
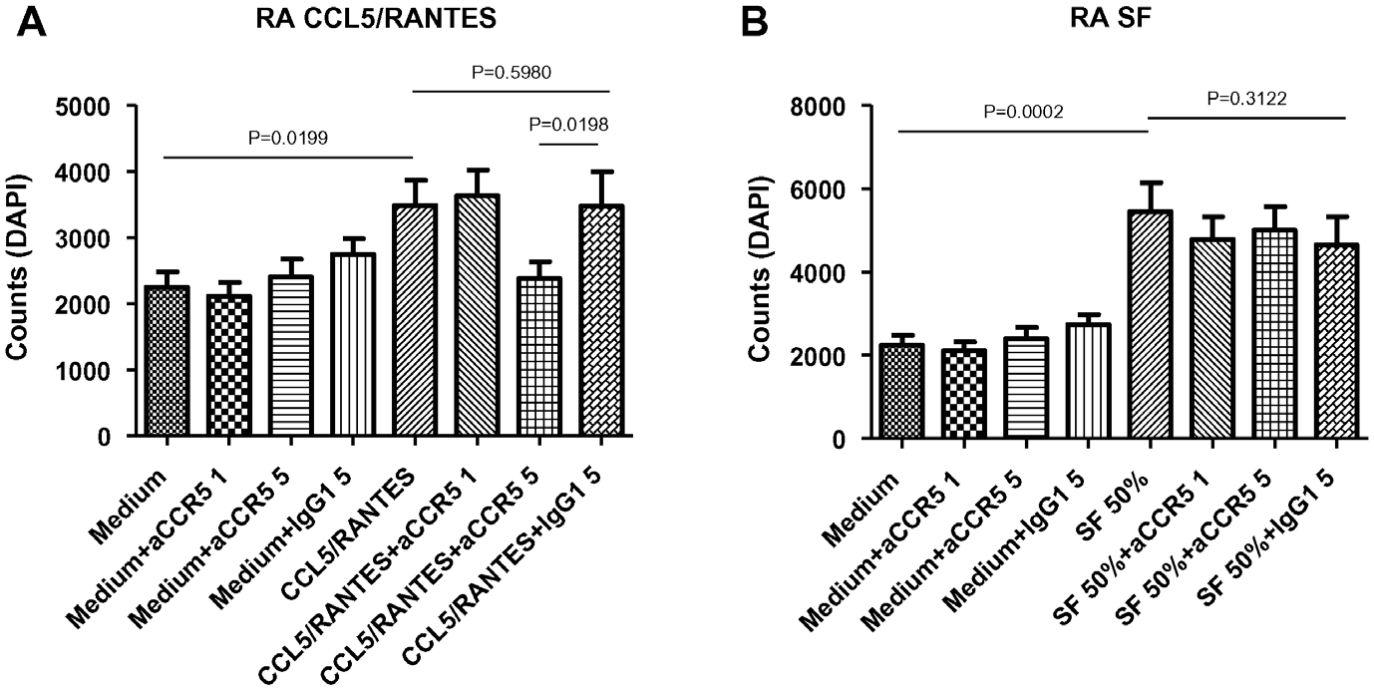
Anti-CCR5 blocks CCL5/RANTES- but not SF-induced RA monocyte migration. (A) RA monocyte migration induced by CCL5/RANTES. (B) RA monocyte migration induced by SF. Data are expressed as mean ± SEM (n = 8).

CCL5/RANTES induced significant migration of RA monocytes (Fig. 2A) pre-incubated with medium (RA P = 0.0199). Anti-CCR5 antibody treatment also blocked CCL5/RANTES-induced migration of RA monocytes (5 µg/ml, P = 0.0198 compared to isotype control).

While SF induced significant migration of RA monocytes (Fig. 2B) pre-incubated with medium (RA P<0.0002), this migration could not be blocked by anti-CCR5 antibody treatment.

### Dual targeting of CCR2 and CCR5 does not significantly block SF-induced RA monocyte migration

**Figure 3:**
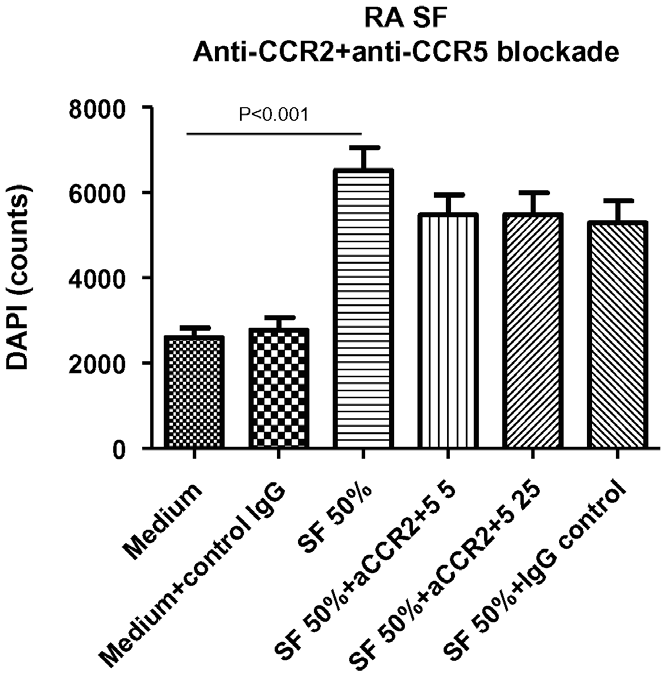
Dual targeting of CCR2 and CCR5 does not block SF-induced RA monocyte migration. Data are expressed as mean ± SEM (n = 6).

Having shown that blockade of either CCR2 or CCR5 could not inibit SF-induced chemotaxis, we investigated whether blockade of both chemokine receptors in combination would result in effective inhibition of monocyte migration towards SF. However, combined blockade of CCR2 and CCR5 did not significantly inhibit SF-induced chemotaxis, even at the concentration of 25 µg/ml (Figure 3).

### CCR1 blockade inhibits both CCL5/RANTES- and SF-induced RA monocyte migration

**Figure 4:**
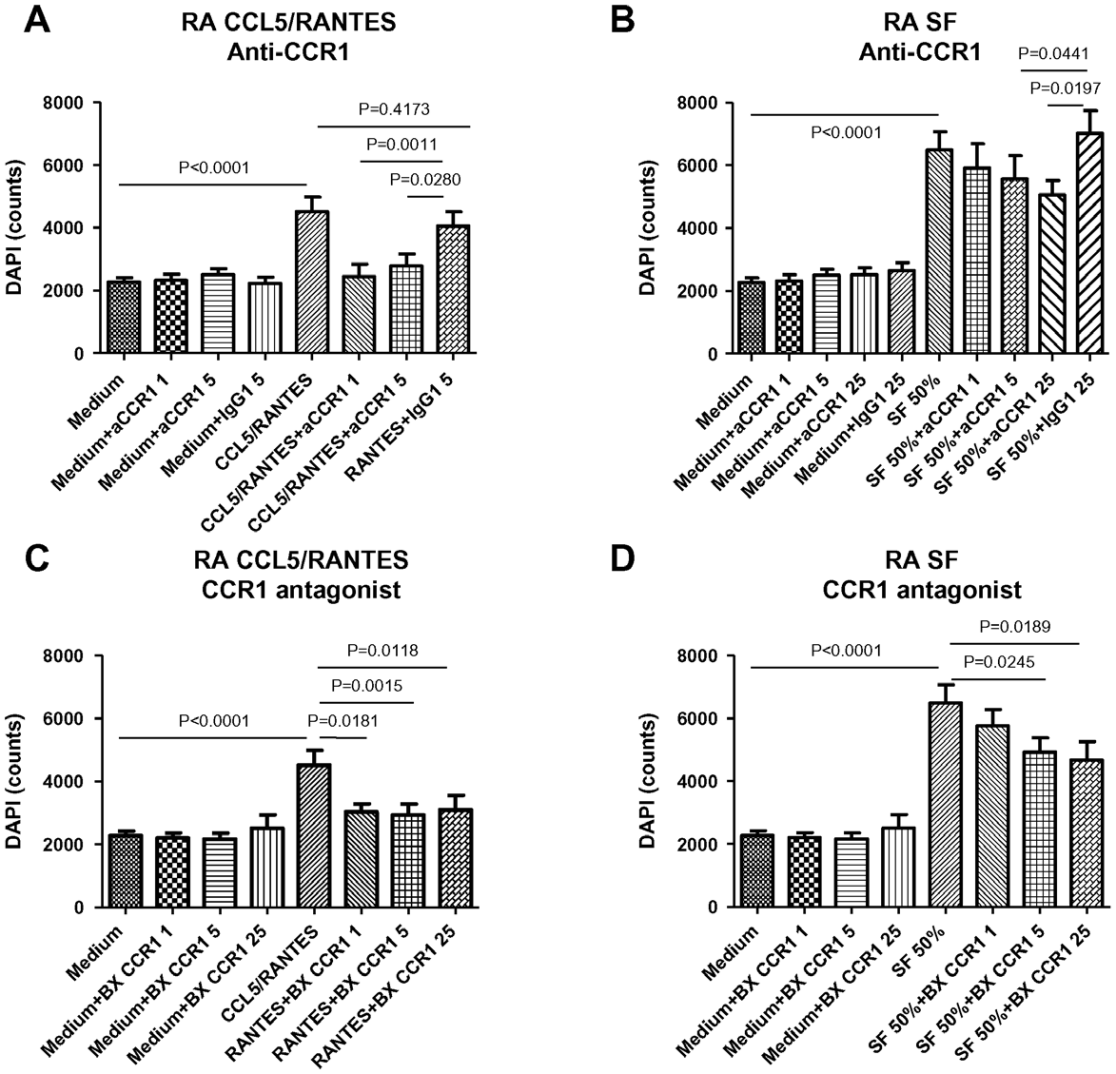
CCR1 blockade inhibits both CCL5/RANTES- and SF-induced RA monocyte migration (A) RA monocyte migration induced by CCL5/RANTES and blocked by anti-CCR1 antibody. (B) RA monocyte migration induced by SF and blocked by anti-CCR1 antibody. (C) RA monocyte migration induced by CCL5/RANTES and blocked by small molecule CCR1 antagonist (BX471). (D) RA monocyte migration induced by SF and blocked by small molecule CCR1 antagonist (BX471). Data are expressed as mean ± SEM (n = 8).

As receptors expressed by monocytes other than CCR2 and CCR5 might also be involved in the recruitment of monocytes to the inflamed synovial compartment, we explored whether CCR1 blockade might result in inhibition of CCL5/RANTES- and/or SF-induced RA monocyte migration. Anti-CCR1 antibody treatment blocked CCL5/RANTES-induced migration of RA monocytes (1 µg/ml, P = 0.0011; 5 µg/ml, P = 0.0280 compared to isotype control) (Fig. 4A). Similarly, the small molecule CCR1 antagonist BX471 also blocked CCL5/RANTES-induced migration of RA monocytes (1 µg/ml, P = 0.0181; 5 µg/ml, P = 0.0015; 25 µg/ml, P = 0.0118 compared to CCL5/RANTES) (Fig. 4C). In contrast to CCR2 or CCR5 blockade, CCR1 blockade resulted in significant reduction of monocyte migration towards SF (Fig. 4B and 4D) (for anti-CCR1: 5 µg/ml, P = 0.0441; 25 µg/ml, P = 0.0197 compared to isotype control; for small molecule CCR1 antagonist: 5 µg/ml, P = 0.0245; 25 µg/ml, P = 0.0189 compared to CCL5/RANTES).

## Discussion

In the present study we showed that specifically for CCR2 and CCR5 blockade, ligand-induced RA peripheral blood monocyte migration could be blocked by the respective receptor blocking antibody (CCL2: anti-CCR2 or CCL5: anti-CCR5) but not when RA SF was used as chemoattractant. Similarly, combined blockade of CCR2 and CCR5 could not significantly inhibit migration of RA peripheral blood monocytes towards SF in the in vitro chemotaxis model. In contrast, we were able to block CCR1-mediated monocyte migration induced by CCL5/RANTES or SF by using either a CCR1 blocking antibody or a small molecule CCR1 antagonist.

We focused in this study on monocytes, as it has previously been shown that numbers of monocytes/macrophages are related to clinical signs and symptoms in RA [3]. Moreover, effective anti-rheumatic treatments in RA induce a decrease in numbers of synovial sublining macrophages, which correlate with clinical improvement independently of the therapeutic strategy (reviewed in [26]). If CCR2 or CCR5 blockade would work in RA, it should be at least in part be via an effect on monocyte migration towards the synovial compartment.

Chemokines and their receptors have been shown to participate in a number of various biological processes and due to their diverse role in autoimmune diseases have been considered good therapeutic targets, in particular CCR2 and CCR5 for immune-mediated inflammatory diseases of which RA is a prototype disease [27]–[29]. In view of these observations, a number of chemokine receptor antagonists (small molecule receptor antagonists and neutralizing antibodies to the receptor) have been designed and tested in animal models and several clinical trials [27], [30]. While CCR2 and CCR5 receptor antagonists have shown initial promise in pre-clinical studies [14], [15], [27], blockade of CCR2 [16], its ligand CCL2 [31], and CCR5 [17], [18] have failed in clinical trials in RA patients [16]–[18]. The picture may be different for CCR1 blockade. In a small, randomized clinical trial, patients with active RA were treated for 2 weeks with the CCR1 antagonist CP-481,715 or placebo. In this proof-of-concept study, treatment was administered with a dose of 300 mg given every eight hours. Synovial tissue analysis revealed a marked decrease in the total number of cells, especially in the number of macrophages and CCR1^+^ cells, after active treatment; only cells capable of expressing CCR1 were affected [21]. The biological changes were associated with a trend towards clinical improvement. A larger phase II clinical trial used a reformulated version of CP-481,715 that was dosed twice a day. This study failed to demonstrate clinical efficacy, which may be related to lower drug levels and very high placebo responses in this study [23], [32]. Another study comparing the effects of another oral CCR1 antagonist, MLN3897, at a dose of 10 mg once daily also failed to demonstrate clinical efficacy compared to placebo at the levels of receptor occupancy reached in that study [22].

There may be different explanations for the negative results in the clinical trials. First, we cannot completely exclude the possibility that the levels receptor occupancy needed to effectively block the CCR2 or CCR5 were not achieved in the clinical trials. However, the results presented here suggest that RA peripheral blood monocyte migration towards RA SF cannot be effectively blocked by targeting CCR2 or CCR5, as other chemokine receptors may be more important. Second, CCR5 is expressed by T regulatory cells in humans [33]. Therefore, the lack of efficacy of treatment with a CCR5 antagonist could perhaps in part be explained by inhibition of T regulatory cells. This may also be relevant for the observation with the CCR2 antagonists, as CCR2 and CCR5 are very close in homology, and inhibitors often target both [34].

Apart from these and other mechanisms, it has been suggested that redundancy in the chemokine system may explain the failed trials with CCR2 and CCR5 antagonists. We found that SF-induced monocyte chemotaxis was not affected when one chemokine receptor was blocked, opposed to ligand (CCL2 or CCL5)-induced monocyte chemotaxis. As in RA patients the synovial joint (tissue and SF) contains several ligands for both CCR2 and CCR5 [1], [8]–[13] that are responsible for monocyte recruitment to these compartments (via many receptors such as CCR1 [21]), this redundancy could have accounted for the observed chemokine receptor blockade failure in both our *in vitro* model and in the clinical trials. However, even when CCR2 and CCR5 were blocked simultaneously (similarly at high doses), we were not able to block SF-induced chemotaxis of RA peripheral blood monocytes, suggesting that CCR2 and CCR5 may not be the crucial chemokine receptors promoting monocyte migration towards the inflamed joint. We also might consider other possible explanations for the lack of clinical improvement after blockade of CCR2 or CCR5. In this respect we have showed by monocyte scintigraphy that blocking only the influx of inflammatory cells may be insufficient to induce clinical improvement [35], and it may be important to target chemokine receptors that also interfere with macrophage retention.

Our results suggest that, in contrast to blockade of CCR2 or CCR5, blocking CCR1 may be sufficient to inhibit migration of RA peripheral blood monocytes towards the inflamed synovial compartment in RA. It is conceivable that high levels of CCR1 occupancy at all times are needed to induce clinical improvement in RA, consistent with our original observations using 300 mg of CP-481,715 every eight hours in RA patients [21].

In summary, CCR2 and CCR5 antagonism may have failed in RA due to redundancy: other chemokine receptors may have substituted for CCR2 and CCR5. In contrast, CCR1 blockade may be sufficient to inhibit migration of RA peripheral blood monocytes towards the synovial compartment in the continuous presence of high levels of receptor occupancy. This notion is supported by recent modeling studies [36].
